# Bibliometric evaluation of Forensic Science International as a scholarly journal within the subject category legal medicine

**DOI:** 10.1016/j.fsisyn.2023.100438

**Published:** 2023-09-16

**Authors:** Alan Wayne Jones

**Affiliations:** Division of Clinical Chemistry and Pharmacology, Department of Biomedical and Clinical Sciences, Faculty of Medicine and Health Sciences, University of Linköping, Linköping, SE-58183, Sweden

**Keywords:** Authorship, Bibliometrics, Citation analysis, Forensic journals, Journal impact factor, Scholarly publishing

## Abstract

This article presents a bibliometric evaluation of **Forensic Science International** (FSI) as a scholarly journal within the “legal medicine” subject category. Citation data were retrieved from Science Citation Index (SCI) and Journal Citation Reports (JCR), both of which are part of the Web-of-Science (WOS) database. The most cited articles in FSI were identified along with the most prolific authors. The current journal impact factor (JIF) of FSI is 2.2, which was in good agreement with the 5-year JIF of 2.3. FSI was ranked fourth among 17 journals within the legal medicine subject category. Since 1979, a total of 209 FSI articles were cited over 100 times and the H-index for times cited was 125. Although widely used in academia, bibliometric methods might also prove useful in jurisprudence, such as when evaluating the research and publications of people proposed as expert witnesses.

## 1 Introduction

Interest in the field of bibliometrics or scientometrics, which deals with quantitative evaluation of scholarly publications, is steadily increasing [1]. Such studies might entail identifying the most cited articles published in a particular scientific journal, the patterns of co-authorship of these articles as well as other citation metrics, all of which are available on-line via the Web-of-Science (WOS) database [[2], [3], [4]].

A veritable pioneer in the field of bibliometrics and information science was Eugene Garfield (1925–2017), who founded the Philadelphia-based organization Institute for Scientific Information in the late 1950s [5,6]. This organization was later acquired by Thomson Reuters and more recently by Clarivate Analytics, who are now responsible for compiling the Science Citation Index (SCI) and Journal Citation Reports (JCR), both of which are key products of the WOS [7].

Elsevier's forensic science flagship journal, **Forensic Science International** (FSI), has been included in JCR since 1979 and belongs to the “legal medicine” subject category. Scientific journals are often compared and contrasted in terms of their journal impact factor (JIF). Although JIFs were ostensibly created to help university librarians select the best journals to include in their collections, they are sometimes used for other more dubious reasons [8,9]. For example, JIFs are considered important by journal publishers to promote and market their titles, hopefully attracting submission of better quality articles, new subscribers, and advertisers [10,11]. Those journals with the highest JIF are generally considered more influential in their particular subject category and scientists strive to publish their research results and discoveries in these high impact journals.

Publishing papers in journals with high JIF is considered meritorious when people apply for a new research position or promotion (tenure) and also when competing for research funding [[12], [13], [14]]. However, it is important to remember that the JIF applies to the average article in a journal and not a specific article. Furthermore, the frequency distribution of citations to articles appearing in a scientific journal tends to be skewed to the right; a small percentage of the published papers attracting the bulk of the citations, and some articles might never get cited [15,16].

Success in science is often judged by the importance and usefulness of a person's scholarly publications, which are the *sine qua non* for climbing the academic ladder [17,18]. Besides prolific authorship, a person's publications are increasingly being judged by the number of times they are cited in papers penned by other scientists [19]. Indeed, some people include citation counts with each of the articles listed on their CVs as well as the impact factors of the journals where they were published [20].

This article presents a bibliometric evaluation of FSI as a scholarly journal belonging to the subject category “legal medicine.” All papers published in FSI since 1979 were scrutinized to find the top-ten most cited articles, and the names of the authors contributing most articles to FSI, as well as other citation data. Changes in the JIF of FSI were investigated between 1997 and 2022 and the articles that contributed most citations to the latest JIF (2022) were identified.

## 2 Methods

### 2.1 Science citation index

The citation database used to prepare this article is part of the WOS, the Science Citation Index Expanded edition and Journal Citation Reports, both of which became available on-line at the end of June 2023.

The JCR contained information for about 21,500 scholarly journals and these were sub-divided into ∼250 scientific areas or research disciplines. Note that the same journal might be allocated to one or more different disciplines or subject categories. FSI and other forensic journals were found in the legal medicine category, which contained just 17 titles.

SCI was searched using the publication title “Forensic Science International,” which has been indexed since 1979. This search resulted in 12,772 hits up to 1 August 2023. These articles were then sorted in decreasing order of number of times each item had been cited. The title of each article, the names of the authors, the FSI volume, issue, and page numbers as well as year of publication were available. The number of cited references included in each article was also provided and whether this was a review or an original article.

The number of FSI articles cited more than 100 times was determined as was the journal H-index. A journal with H-index of 100 means that it has published 100 articles or reviews each of which were cited at least 100 times.

### 2.2 Journal citation reports

The impact factor of FSI and other citation data were obtained from the latest (2022) version of JCR. This journal was allocated to the legal medicine category which contained 17 journal titles. The impact factor of any journal J in a particular citation year is calculated as follows:

$$J{IF}_{year}=\frac{{C_{year-2}+C}_{year-1}}{{{CI}_{year-2}+\text{CI}}_{year-1}}$$

Year = The particular JCR year, which is not the publication year.
*JIF*_year_ = The impact factor in a specific JCR year, such as 2022.
*C*_year-2_ = Total citations in JCR year to items published in “year – 2.”
*C*_year-1_ = Total citations in JCR year to items published in “year – 1.”
*CI*_year-2_ = Number of citable items in “year – 2.”
*CI*_year-1_ = Number of citable items in “year – 1.”

Note that JIF reflects the average number of citations received by recently published articles in the target journal. It is a ratio between number of citations to recently published articles and the number of citable items over the same time period. The conventional JIF covers a two year time window before the citation year, namely citations in 2022 to articles published in the journal 2020 and 2021.

Also available in JCR was a five year JIF, which considers citations to articles published during the previous five years. A longer citation window might be more appropriate for some journals when research activity is less intense. However, for the 17 journals within the subject category of legal medicine the 2-year and 5-year JIF were highly correlated with a correlation coefficient of r = 0.98.

Note that the numerator in the JIF formula includes citations to all material published in a particular journal, including self-citations, whereas the denominator only includes the number of substantive items published, defined as research articles, reviews and short communications. The designation as a citable item can sometimes skew the JIF calculation, such as if letters-to-the-editor and or news items and other editorial material happen to become highly cited [21].

### 2.3 Evaluating self-citations

Self-citations occur when a journal article references articles from the same journal and this is a common practice in many branches of scientific publishing. Indeed, self-citations can often make up a significant number of all citations a journal gives and receives in a particular citation year. The self-citing rate of a journal is expressed as a percentage and calculated as the number of references to the journal (FSI) in its own articles to the total number of cites given out by the same journal. The self-cited rate is calculated in a similar way as the number of references in the journal to its own articles expressed as a percentage of the total number of cites received by that journal.

## 3 Results

### 3.1 Characteristics of document type

The 13,740 items published in FSI since 1979 are distributed in Table 1 according to the document type. Most items published were original articles (n = 9,971, 72%), but there were also proceedings papers (n = 968, 7%). The latter probably represent published versions of papers presented at various scientific meetings, such as The International Association of Forensic Toxicologists (TIAFT) or the International Association of Forensic Sciences (IAFS).

**Table 1:** Classification of the documents published in FSI between 1979 and 2022.

Document type	N (%)
Original articles	9,972 (72)
Meeting abstracts	1,778 (13)
Proceedings papers	968 (7)
Editorial material	364 (2.6)
Review articles	306 (2.2)
Letter to editor	224 (1.6)
Correction	95 (0.69)
Other material a	10 (0.07)

a Obituaries, book review, biographical items.

Among document types, there were n = 306 (2.2%) review articles published in FSI, and 95 letters-to-the-editor (0.7%). On two occasions special supplements of FSI were published containing abstracts of conference papers from Triannual Meeting of the International Association of Forensic Sciences (IAFS) in 2017 and Third European Academy of Forensic Sciences meeting in 2003.

### 3.2 Growth in number of items published in FSI

**Fig. 1:**
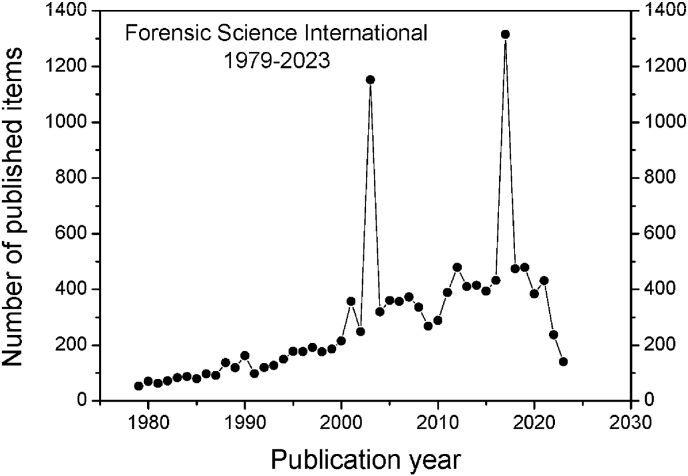
Trends in number of items published in FSI between 1979 and 2023 (1-8-2023). The two spikes in the trend line are explained by supplements containing abstracts of papers presented at forensic conferences. Note that publication count for 2023 is not yet complete.

Fig. 1 shows year-by-year changes in number of items published in FSI between 1979 and 2023. The two spikes in this graph corresponding to the years 2003 and 2017 when as already mentioned FSI published supplements containing the abstracts of conference papers, which are rarely if ever cited.

Otherwise, the number of citable items in FSI has gradually increased since 1979 with a low of 53 in its first publication year increasing to a peak of 479 items in 2019 (omitting the aforementioned conference abstract years). During the first five publication years of FSI (1979–1983) there were 341 substantive items published, which compares with 2005 items during the most recent five year period 2018–2022, hence a 5.8 fold increase.

### 3.3 Most highly cited FSI publications

Table 2 lists the top-ten most highly papers articles published in FSI between 1979 and 2022 showing publication year, total citations, number of authors, surname of first and last author, citations per author, and citation impact of the article (citations/year). Topping the list was a methods paper by Peters et al. [22], which presented guidelines for development and evaluation of new analytical methods in forensic toxicology laboratories. Basic principles of method validation include documentation of accuracy, precision, linearity, LOD and LOQ etc., were described in a pedagogic way, which probably accounts for the high rate of citation, 63.1 cites/year. A single author paper by Wennig [23], which dealt with problems and pitfalls in hair analysis for drugs, also attracted many citations (n = 439).

**Table 2:** Top-ten most highly cited articles published in FSI, pattern of co-authorship, and citation impact.

Cited article title {reference]	Publication year	Total cites	Number of authors	First/last author	Cites per author	Cites per year^a^
Validation of new methods [22]	2007	1013	3	Peters/Musshof	337	63.1
Society of Hair Testing guidelines for drug testing in hair [24]	2012	477	3	Cooper/Kintz	159	43.6
Metal and metalloid multi-elementary ICP-MS validation in whole blood, plasma, urine and hair - Reference values [25]	2005	461	8	Goulle/Lacroix	57	25.6
Potential problems with the interpretation of hair analysis results [23]	2000	439	1	Wennig	439	19.1
Analysis of body fluids for forensic purposes: From laboratory testing to non-destructive rapid confirmatory identification at a crime scene [26]	2009	422	2	Virkler/Lednev	211	30.1
An investigation of the rigor of interpretation rules for STRs derived from less than 100 pg of DNA [27]	2000	422	5	Gill/Buckleton	84	18.3
The problem of aging human remains and living individuals: A review [28]	2009	384	6	Cunha/Cattaneo	64	27.4
Factors affecting decomposition and Diptera colonization [29]	2001	366	3	Campobasso/Introna	122	16.6
Publication of population data of human polymorphisms [30]	2000	349	2	Lincoln/Carracedo	116	15.1
Review: The physiology of saliva and transfer of drugs into saliva [31]	2005	328	2	Aps/Martens	164	18.2

a From publication year until 1-8-2023.

Two highly cited papers dealt with guidelines for analysis and interpretation of drugs in hair strands [23,24], which over the past 30 y has emerged as a popular subject in forensic toxicology. Other examples of highly cited FSI articles making the top-ten dealt with anthropology [28], criminalistics [26], and DNA/genetics [27]. Review articles tend to attract a lot of citations and the 10th most cited article was a comprehensive overview of passage of drugs from blood into saliva [31].

### 3.4 Organizations and countries making most recent contributions

Table 3 lists the top-ten most prolific organizations publishing articles in FSI during the past three year period, with University of Lausanne in Switzerland topping the list. Interestingly, each of the top-10 organizations/universities were from various countries in Europe.

**Table 3:** Top-ten organizations/universities contributing articles to FSI in past three years.

Rank	Organization/university	Country	Number of items
1	University Lausanne	Switzerland	45
2	Udice-French Research Universities	France	34
3	University Technology Sydney	Australia	25
4	Netherlands Forensic Science Institute	Netherlands	24
5	Centre National de la Recherche Scientifique (CNRS)	France	23
6	University Milan	Italy	23
7	University Amsterdam	Netherlands	19
8	University Copenhagen	Denmark	17
9	Swedish National Board of Forensic Medicine	Sweden	16
10	University London	UK	16

Likewise, Table 4 lists the top-ten countries contributing most articles to FSI over the same three year period and forensic practitioners from USA, Australia, and mainland China contributed most articles. There might be a connection between number of contributions and the number of practicing forensic scientists in these nations.

**Table 4:** Top-ten countries contributing most articles to FSI in past three years.

Rank	Country	Number of items
1	USA	173
2	Australia	97
3	Mainland China	95
4	United Kingdom	89
5	Italy	85
6	Switzerland	85
7	Germany	62
8	France	51
9	Canada	50
10	Netherlands	50

### 3.5 Most prolific authors contributing articles to FSI

Table 5 lists the top-ten most prolific authors publishing papers in FSI, the organizations and/or universities they belonged to and countries of residence. Topping the list was Professor Burkhard Madea from the University of Bonn (Germany), who contributing 171 items to FSI between the years 1979 and 2023. Two other major contributors were from Australia (Drummer and Lennard), three from Switzerland (Roux, Margot, and Esseiva) and one from USA (Budowle).

**Table 5:** Top-ten most prolific contributors to papers published in FSI 1979–2023.

Rank	Contributor	Organization or University	Country	FSI articles 1979–2023
1	Madea, B.	University Bonn	Germany	171
2	Roux, CP.	University Lausanne	Switzerland	123
3	Carracedo, A.	University Santiago de Compostela	Spain	78
4	Kintz, P.	University Strasbourg	France	67
5	Budowle, B.	University North Texas, Health Science Center	USA	55
6	Margot, P.	University Lausanne	Switzerland	48
7	Lennard, C.	University Western Sydney	Australia	48
8	Drummer, O.	Monash University	Australia	47
9	Cattaneo, C.	University Milan	Italy	44
10	Esseiva, P.	University Lausanne	Switzerland	43

### 3.6 Changes in the impact factor of FSI over time

**Fig. 2:**
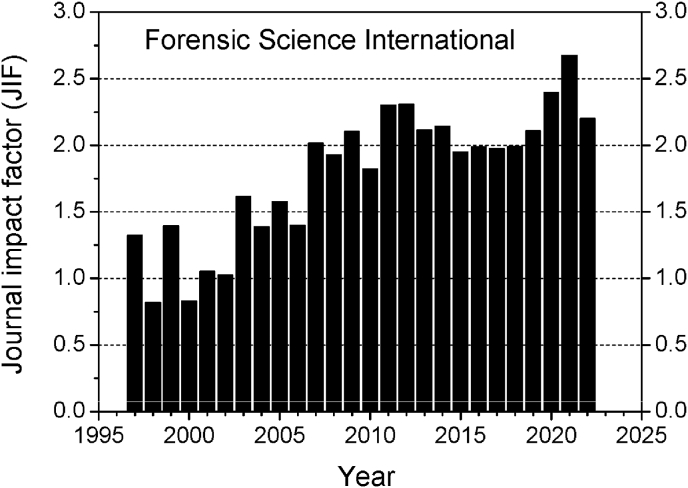
Changes in the impact factor of FSI between 1997 and 2022.

Fig. 2 shows how the impact factor of FSI varied between 1997 and 2022. This graph shows that JIF has almost doubled over this time period. Between 1979 and 2006, JIF was just over 1.0 increasing thereafter to exceed 2.0, which was maintained for several years. According to the latest version of JCR, FSI's 2-year impact factor was 2.2, which aligned well with the 5-year JIF of 2.3. FSI was ranked fourth among the 17 journals within the legal medicine subject category. A JIF of 2.0 means that the average article published in FSI was cited twice in the two year window after the publication year.

In the citation year 2022, FSI received 920 citations to articles published in 2020 and 787 citations to articles published in 2021 from all journals in the database. This makes a total of 1707 citations for inclusion in the impact factor calculation (see methods section). During the same two years (2020 and 2021), FSI published 370 and 405 citable items, respectively (total 775 items). The JIF for 2022 is therefore calculated as 1707/775 = 2.2. However, of the 1707 citations, 152 were self-citations (8.9%), so the JIF after correcting for self-citations was 2.0 (1555/775 = 2.0).

Table 6 lists the articles published in FSI that contributed 10 or more citations to the 2022 JIF calculation. Surnames of first and last author on these articles are shown along with the number of citations to FSI articles in the relevant time window. Most citations to FSI were from articles published in the same journal, hence self-citations (n = 152). Another 64 cites came from articles published in Journal of Forensic Science, 57 cites from International Journal of Legal Medicine, 42 from WIRE's Forensic Science, 39 from Science & Justice and 30 from Journal of Analytical Toxicology.

**Table 6:** Articles published in FSI that contributed 10 or more citations towards the 2022 journal impact factor.

Rank	Article title [reference]	Year published	First/last author	Citation count
1	Violence against women in the Covid-19 pandemic: A review of the literature and a call for shared strategies to tackle health and social emergencies [32]	2021	Viero/Cattaneo	32
2	Dimensionality reduction and visualisation of hyperspectral ink data using t-SNE [33]	2020	Devassy/George	26
3	A DWT-SVD based robust digital watermarking for medical image security [34]	2021	Zermi/Euschi	23
4	Fatal poisoning in drug addicts in the Nordic countries in 2017 [35]	2020	Simonsen/Ojanpera	16
5	Recent advances in digital image manipulation detection techniques: A brief review [36]	2020	Thakur/Rohilla	15
6	Concentrations of THC, CBD, and CBN in commercial hemp seeds and hempseed oil sold in Korea [37]	2020	Jang/Han	13
7	Alcohol and illicit substances associated with fatal crashes in Queensland: An examination of the 2011 to 2015 Coroner's findings [38]	2020	Davey/Parkes	11
8	Isotonitazene: Fatal intoxication in three cases involving this unreported novel psychoactive substance in Switzerland [39]	2021	Mueller/Grata	11
9	Automated face recognition in forensic science: Review and perspectives [40]	2020	Jacquet/Champod	10
10	Automated latent fingerprint identification system: A review [41]	2020	Singla/Sofat	10
11	Cannabidiol and tetrahydrocannabinol concentrations in commercially available CBD E-liquids in Switzerland [42]	2020	Grafinger/Weinmann	10
12	Fatal methemoglobinemia: A case series highlighting a new trend in intentional sodium nitrite or sodium nitrate ingestion as a method of suicide [43]	2021	Hickey/Pickup	10
13	Providing illicit drugs results in 5 s using ultra-portable NIR technology: An opportunity for forensic laboratories to cope with the trend toward the decentralization of forensic capabilities [44]	2020	Coppey/Esseiva	10
14	Vitreous humor endogenous compounds analysis for post-mortem forensic investigation [45]	2020	Pigaiani/Tagliaro	10

### 3.7 Comparison of cited vs citing journals

Table 7 lists the top eight journals that cited papers published in FSI (FSI cited journal) along with the top journals that FSI articles cited (FSI citing journal). There were many more citations to articles published in FSI (n = 14,958) compared with citations from FSI to other journals in the database (n = 8,921). Note that of all citations to FSI articles, 958 were self-citations.

**Table 7:** The top-eight journals most frequently cited by FSI articles (citing journal) and the top-eight journals that most often cited FSI articles (cited journal) in 2022.

Citing journal	N (%)	Cited journal	N (%)
Forensic Sci Int	958 (6.4)^a^	Forensic Sci Int	958 (10.7)^b^
Int J Legal Med	608 (4.1)	J Forensic Sci	452 (5.1)
J Forensic Sci	454 (3.0)	Int J Legal Med	245 (2.7)
WIRE's Forensic Sci	327 (2.2)	Forensic Sci Int Gen	198 (2.2)
Sci & Justice	326 (2.2)	Sci & Justice	137 (1.5)
Legal Med	294 (2.0)	Am J Phys Anthropol	103 (1.1)
J Anal Tox	276 (1.8)	J Forensic Leg Med	86 (0.9)
Drug Test Anal	225 (1.5)	J Anal Tox	62 (0.7)
Citations to FSI articles from all journals	14,958^c^	Citations from FSI articles to all journals	8,921^d^

a Percent of all references to FSI in its own articles to all cites given out by FSI (self-citing rate).

b Percent of references to FSI in its own articles to total cites received by FSI (self-cited rate).

c Citations to FSI articles from all journals included in the database.

d Citations from FSI articles to all journals in the database.

## 4 Discussion

Forensic science is a multidisciplinary topic requiring knowledge of medicine, science, and technology and the gathering, analysis, and the interpretation of evidence in a legal context [46,47]. Scientific evidence is often crucial in many types of criminal and civil litigation and both defence and prosecution attorneys have the opportunity to consult and retain expert witness to present and explain scientific evidence in court [48]. The main task of the expert is to interpret the scientific and technical evidence to a judge and jury and point out the strengths and weaknesses, when the guilt or innocence of a suspect is decided [49].

An expert witness needs to have the appropriate education, skill, and training above and beyond the ken of the average lay person (jury member). When scientific evidence is presented in court, an important thing to consider is whether the work has been subjected to peer review and publication [50]. In this context, it might also be relevant to know the journal's impact factor and how many times the article in question was cited by other scientists since it appeared in print. Highly cited papers are generally considered more authoritative than articles that are seldom or never cited [51].

Bibliometric methods might prove useful to verify the qualifications of people proposed as expert witnesses, such as to check their current research activity and whether they have contributed articles on the topic being litigated in a specific case. Obviously, the opinions of people with a strong publication and citation record should be given more credibility than experts that don't contribute to the scientific literature at all. If the journal article proffered in evidence has garnered many citations, this is one indication the work in question has been generally accepted by the relevant scientific community [51].

Many bibliometric studies have been done to investigate journals specializing in a wide range of scientific disciplines, including criminalistics [[52], [53], [54]], but little attention has hitherto been given to forensic science and legal medicine journals. A publicly available citation database was recently used to investigate the most highly cited forensic scientists in the United States [55] and the Nordic countries [56].

Quality is not the same as quantity when it comes to scholarly publications [57,58].The best way to judge the importance and usefulness of a scientific paper is to read the article in question and decide in what way it might influence your own research endeavours. A widely used surrogate for quality is the number of times an article is cited, which in a nutshell is why citation databases have become so popular in research assessment exercises [59].

The most cited authors are not necessarily the most prolific authors, although a strong association exists between the two [60]. A proxy for the quality and prestige of a scientific journal within its subject discipline is the JIF, although this should never be used in isolation [61]. JIF defines the frequency with which the average article in a journal is cited in a particular year or time period and not a specific article [62]. A high JIF might be attributed to just a few highly cited articles in that journal [63], whereas the vast majority might never be cited, not even a self-citation [64,65].

The concept and meaning of the impact factor of forensic journals was discussed in comparison with the JIF of journals specializing in basic sciences and non-forensic disciplines [66]. Ten years later, in a follow-up paper, forensic science and toxicology journals were compared and contrasted in terms of their JIF and some suggestions were made on how best to interpret this citation metric [67].

Self-citations can skew the JIF calculation, although in the most recent versions of JCR this metric is available with and without including self-citations [68]. Self-citation rates can differ widely between different journals and disciplines and excessive self-citation is frowned upon and considered a way to manipulate the JIF calculation. The high self-citation rate by FSI's sister journal, FSI Genetics, led to it being temporarily excluded from JCR in 2019 [69]. The editors made a written complaint to Clarivate Analytics and after re-consideration FSI Genetics was reinstated. In the citation year 2022, the JIF of FSI genetics was 3.1, but when self-citations (n = 325 or 34.5%) were removed from the calculation, the JIF dropped to 2.1, which compares with 152 self-citations for FSI (8.9%).

In conclusion, this article is the first to make an in-depth evaluation of the citation metrics of FSI by identifying the number of articles published, the most highly cited articles and the most prolific authors of papers appearing in FSI. Hopefully, this article will stimulate interest among forensic practitioners for further bibliometric studies and the quantitative evaluation of scholarly publications. Besides obvious applications in academia and library sciences, bibliometric can be used to find the most influential articles on a particular topic (most cited) and also to verify the qualifications of people proposed to serve as expert witnesses.

## Funding sources

There was no external funding applied for nor received to prepare this article.

## Declaration of competing interest

The author of this article does not consider there are any conflicts of interest in submitting this work for peer review and publication.

## References

[1] Blockmans W., Engwall L., Weaire D. (2014). Bibliometrics: use and abuse in the review of research performance. Portland Press, Weener Gren International Series.

[2] Garfield E. (1955). Citation indexes for science; a new dimension in documentation through association of ideas. Science.

[3] Garfield E. (1964). "Science citation index"--A new dimension in indexing. Science.

[4] Garfield E. (1972). Citation analysis as a tool in journal evaluation. Science.

[5] Wouters P. (2017). Eugene Garfield (1925-2017). Nature.

[6] Schoenbach U.H., Garfield E. (1956). Citation indexes for science. Science.

[7] Adam D. (2002). The counting house. Nature.

[8] Garfield E. (2006). The history and meaning of the journal impact factor. JAMA, J. Am. Med. Assoc..

[9] Garfield E. (2001). Interview with Eugene Garfield, chairman emeritus of the Institute for scientific information (ISI), cortex. a journal devoted to the study of the nervous system and behavior.

[10] Garfield E. (2001). Impact factors, and why they won't go away. Nature.

[11] Garfield E. (1999). Journal impact factor: a brief review. CMAJ (Can. Med. Assoc. J.) : Canadian Medical Association journal = journal de l'Association medicale canadienne.

[12] Seglen P.O. (1989). From bad to worse: evaluation by Journal Impact. Trends Biochem. Sci..

[13] Seglen P.O. (1997). Citations and journal impact factors: questionable indicators of research quality. Allergy.

[14] Seglen P.O. (1997). Why the impact factor of journals should not be used for evaluating research. BMJ.

[15] Dimitrov J.D., Kaveri S.V., Bayry J. (2010). Metrics: journal's impact factor skewed by a single paper. Nature.

[16] Smart P. (2015). Is the impact factor the only game in town?. Ann. R. Coll. Surg. Engl..

[17] Moed H.F. (2009). New developments in the use of citation analysis in research evaluation. Arch. Immunol. Ther. Exp..

[18] Ioannidis J.P., Boyack K.W. (2020). Citation metrics for appraising scientists: misuse, gaming and proper use. Med. J. Aust..

[19] Tomlinson S. (2000). The research assessment exercise and medical research. Br. Med. J..

[20] Ioannidis J.P., Boyack K.W., Small H., Sorensen A.A., Klavans R. (2014). Bibliometrics: is your most cited work your best?. Nature.

[21] Jones A.W. (2005). Mode of classification of source material as citable items skews journal impact factor calculations. Scand. J. Clin. Lab. Invest..

[22] Peters F.T., Drummer O.H., Musshoff F. (2007). Validation of new methods. Forensic Sci. Int..

[23] Wennig R. (2000). Potential problems with the interpretation of hair analysis results. Forensic Sci. Int..

[24] Cooper G.A., Kronstrand R., Kintz P., Society of Hair T. (2012). Society of Hair Testing guidelines for drug testing in hair. Forensic Sci. Int..

[25] Goulle J.P., Mahieu L., Castermant J., Neveu N., Bonneau L., Laine G., Bouige D., Lacroix C. (2005). Metal and metalloid multi-elementary ICP-MS validation in whole blood, plasma, urine and hair. Reference values, Forensic Sci. Int..

[26] Virkler K., Lednev I.K. (2009). Analysis of body fluids for forensic purposes: from laboratory testing to non-destructive rapid confirmatory identification at a crime scene. Forensic Sci. Int..

[27] Gill P., Whitaker J., Flaxman C., Brown N., Buckleton J. (2000). An investigation of the rigor of interpretation rules for STRs derived from less than 100 pg of DNA. Forensic Sci. Int..

[28] Cunha E., Baccino E., Martrille L., Ramsthaler F., Prieto J., Schuliar Y., Lynnerup N., Cattaneo C. (2009). The problem of aging human remains and living individuals: a review. Forensic Sci. Int..

[29] Campobasso C.P., Di Vella G., Introna F. (2001). Factors affecting decomposition and Diptera colonization. Forensic Sci. Int..

[30] Lincoln P., Carracedo A. (2000). Publication of population data of human polymorphisms. Forensic Sci. Int..

[31] Aps J.K., Martens L.C. (2005). Review: the physiology of saliva and transfer of drugs into saliva. Forensic Sci. Int..

[32] Viero A., Barbara G., Montisci M., Kustermann K., Cattaneo C. (2021). Violence against women in the Covid-19 pandemic: a review of the literature and a call for shared strategies to tackle health and social emergencies. Forensic Sci. Int..

[33] Devassy B.M., George S. (2020). Dimensionality reduction and visualisation of hyperspectral ink data using t-SNE. Forensic Sci. Int..

[34] Zermi N., Khaldi A., Kafi R., Kahlessenane F., Euschi S. (2021). A DWT-SVD based robust digital watermarking for medical image security. Forensic Sci. Int..

[35] Simonsen K.W., Kriikku P., Thelander G., Edvardsen H.M.E., Thordardottir S., Andersen C.U., Jonsson A.K., Frost J., Christoffersen D.J., Delaveris G.J.M., Ojanpera I. (2020). Fatal poisoning in drug addicts in the Nordic countries in 2017. Forensic Sci. Int..

[36] Thakur R., Rohilla R. (2020). Recent advances in digital image manipulation detection techniques: a brief review. Forensic Sci. Int..

[37] Jang E., Kim H., Jang S., Lee J., Baeck, S S., Kim E., Kim Y.U., Han E. (2020). http://www.ncbi.nlm.nih.gov/pubmed/31786513.

[38] Davey J.D., Armstrong K.A., Freeman J.E., Parkes A. (2020). Alcohol and illicit substances associated with fatal crashes in Queensland: an examination of the 2011 to 2015 Coroner's findings. Forensic Sci. Int..

[39] Mueller F., Bogdal C., Pfeiffer B., Andrello L., Ceschi A., Thomas A., Grata E. (2021). Isotonitazene: fatal intoxication in three cases involving this unreported novel psychoactive substance in Switzerland. Forensic Sci. Int..

[40] Jacquet M., Champod C. (2020). Automated face recognition in forensic science: review and perspectives. Forensic Sci. Int..

[41] Singla N., Kaur M., Sofat S. (2020). Automated latent fingerprint identification system: a review. Forensic Sci. Int..

[42] Grafinger K.E., Kronert S., Broillet A., Weinmann W. (2020). Cannabidiol and tetrahydrocannabinol concentrations in commercially available CBD E-liquids in Switzerland. Forensic Sci. Int..

[43] Hickey T.B.M., MacNeil J.A., Hansmeyer C., Pickup M.J. (2021). Fatal methemoglobinemia: a case series highlighting a new trend in intentional sodium nitrite or sodium nitrate ingestion as a method of suicide. Forensic Sci. Int..

[44] Coppey F., Becue A., Sacre P.Y., Ziemons E.M., Hubert P., Esseiva P. (2020). Providing illicit drugs results in five seconds using ultra-portable NIR technology: an opportunity for forensic laboratories to cope with the trend toward the decentralization of forensic capabilities. Forensic Sci. Int..

[45] Pigaiani N., Bertaso A., De Palo E.F., Bortolotti F., Tagliaro F. (2020). Vitreous humor endogenous compounds analysis for post-mortem forensic investigation. Forensic Sci. Int..

[46] Clark M.J. (2005). Forensic. Lancet.

[47] O'Brien E., Nic Daeid N., Black S. (2015). Science in the court: pitfalls, challenges and solutions. Phil. Trans. Roy. Soc. Lond. B Biol. Sci..

[48] Milroy C.M. (2017). http://www.ncbi.nlm.nih.gov/pubmed/31240003.

[49] Stern H.S., Cuellar m., Kaye (2019). Reliability and validity of forensic science evidence. Significance.

[50] Keierleber J.A., Bohan T.L. (2005). Ten years after Daubert: the status of the states. J. Forensic Sci..

[51] Jones A.W. (2022). Highly cited forensic practitioners in the discipline legal and forensic medicine and the importance of peer-review and publication for admission of expert testimony. Forensic Sci. Med. Pathol..

[52] Cohen E.G., Farrington D.P., Iratzoqui A. (2020). Changes in the most-cited scholars in 20 criminology and criminal justice journals between 1990 and 2015 and comparisons with the Asian journal of criminology. Asian J. Criminol..

[53] Cohn E., A. Iratzoqui G., Farrington D.P., Piquero A., Powell Z.A. (2018). Most-cited articles and authors in crime and justice. Crime Justice.

[54] Cohn E.G., Farrington D.P. (1994). Who are the most-cited scholars in major American criminology and criminal justice journals?. J. Crim. Justice.

[55] Jones A.W. (2023). Who are the most highly cited forensic scientists in the United States?. J. Forensic Sci..

[56] Jones A.W. (2023). Highly cited forensic practitioners in the Nordic countries and their composite citation scores based on six different citation metrics. Med. Leg. J..

[57] Harvey L.A. (2020). We need to value research quality more than quantity. Spinal Cord.

[58] Sahel J.A. (2011). Quality versus quantity: assessing individual research performance. Sci. Transl. Med..

[59] Sandstrom U., van den Besselaar P. (2016). Quantity and/or quality? The importance of publishing many papers. PLoS One.

[60] Huang S.W. (2016). Positive correlation between quality and quantity in academic journals. J. Info..

[61] Woolston C. (2021). Impact factor abandoned by Dutch university in hiring and promotion decisions. Nature.

[62] Walter G., Bloch S., Hunt G., Fisher K. (2003). Counting on citations: a flawed way to measure quality. Med. J. Aust..

[63] Paulus F.M., Cruz N., Krach S. (2018). The impact factor fallacy. Front. Psychol..

[64] Van Noorden R. (2017). The science that's never been cited. Nature.

[65] Chew M., Villanueva E.V., Van Der Weyden M.B. (2007). Life and times of the impact factor: retrospective analysis of trends for seven medical journals (1994-2005) and their Editors' views. J. R. Soc. Med..

[66] Jones A.W. (1993). The impact of forensic science journals. Forensic Sci. Int..

[67] Jones A.W. (2003). Impact factors of forensic science and toxicology journals: what do the numbers really mean?. Forensic Sci. Int..

[68] Van Noorden R., Singh Chawla D. (2019). Hundreds of extreme self-citing scientists revealed in new database. Nature.

[69] Carracedo A., Butler J.M., Gusmao L., Linacre A., Parson W., Schneider P.M., Vallone P.M., Vennemann M. (2020). On the suppression of forensic science international: genetics from the 2019 journal citations report. Forensic Sci. Int. Genet..

